# A multilevel selection model for prosocial well-being

**DOI:** 10.3389/fpsyg.2023.1068119

**Published:** 2023-02-23

**Authors:** Mads Larsen, Nina Witoszek, June Chun Yeung

**Affiliations:** ^1^Centre for Development and the Environment, University of Oslo, Oslo, Norway; ^2^Institute of Psychology, Polish Academy of Sciences, Warsaw, Poland

**Keywords:** happiness, meaning, multilevel selection, Nordic model, prosociality, qualitative interviews, volunteerism, well-being societies

## Abstract

This article proposes an evolutionary model for well-being informed by multilevel selection. We posit that people’s subjective assessment of their own quality of life is the sum their happiness, which is related to individual selection, and their sense of having a meaningful life, which is related to group selection. Conceptualizing life quality as “Happiness + Meaning = Well-being” offers insights into how the human well-being system helps people navigate between individual and group needs. We define happiness as the cluster of affects that reward individuals for solving adaptively relevant problems. We approach meaning as a reward individuals experience when contributing to their community. While people derive happiness from cooperation and competition, meaning originates from prosocial (cooperative/altruistic) behavior. Since increased within-group competition often reduces societal well-being, public policy should aim at cooperative means for good living. Our model brings attention to these dynamics. The Nordic countries, which score highest on quality of life, facilitate multilevel well-being, that is, individual prosperity and altruistic opportunity. Our preliminary quantitative study confirmed the correlation between some markers of prosociality and well-being at a national level. To investigate the psychological mechanisms behind this correlation, we conducted in-depth interviews of Nordic and Slavonic helpers of Ukrainian refugees in Norway (*n* = 32). A primary ambition was to illuminate how the human quest for meaning contributes both to individual flourishing and group selection. In line with Nesse’s view on happiness not as an affect meant to be maximized, but an evolutionary signal, we use a qualitative approach that allows for a deeper understanding of how individuals adapt to these signals. Our findings suggest that happiness is transient so that the well-being system’s signal sensitivity can be preserved. Meaning is enduring since it assesses and reinforces social belonging. These insights are relevant for our era’s turn toward more holistic development policies. Compared to often materialistic, competition-driven happiness pursuits, meaning-driven well-being is a more sustainable alternative for individuals, communities, and the planet.

## 1. Introduction

The 21st-century turn toward increasing human wellbeing as part of the global development agenda calls for policies that facilitate good lives in a sustainable manner. Seeking to uncover what makes humans flourish, positive psychology has become a thriving field, yet Buss and Nesse’s early-2000s calls for an evolutionary approach have largely been disregarded. This article responds to these pleas by synthesizing Buss and Nesse’s works on happiness with Baumeister’s work on meaning. The concepts of happiness and meaning have elicited a plethora of often contradicting definitions in scholarly and popular discourses. We contend that it could be profitable to apply a multilevel selection perspective as a means for transcending some of these contradictions. With the equation Happiness + Meaning = Well-being, we seek to: (1) offer an alternative to positive psychology’s conceptual overabundance, (2) contribute to the field’s move toward greater cultural sensitivity, and (3) illustrate how an evolutionary approach can engender insights with policy implications.

When the United Nations in 2011 unanimously adopted the resolution “Happiness: toward a holistic approach to development,” Secretary-General Ban Ki-moon declared that “while material prosperity is important, it is far from being the only determinant of well-being.” Dozens of countries have since adopted well-being accounts (Diener et al., 2015; Durand, 2018). The UN’s *World Happiness Report* began to rank nations in terms of how citizens assess their own quality of life. This weakening faith in the utility of economic metrics was partially a reaction to the broken promise of Western modernity. Growth and technological advances have to an almost miraculous extent made our lives easier, safer, and more pleasurable. Nesse (2005) points to how the expectation of leading Enlightenment thinkers, that the elimination of suffering “would lead to general happiness is not only unfulfilled, it is almost a cruel joke.” He therefore encourages well-being scholars to incorporate insights from the evolutionary sciences into their models for studying human contentment.

After Diener (1984) popularized the well-being field among psychologists, it has arguably become the hottest topic of social science (De Vos, 2012). A primary purpose has been to recommend policy that can raise well-being for populations (Diener et al., 2009a). In terms of economic growth, this goal has been complicated by the *Easterlin paradox* (Easterlin, 1974; Stevenson and Wolfers, 2008; Hellevik, 2011), which indicates that richer individuals tend to be happier, but that societies do not gain much in happiness once an average income covers basic needs.^1^ Doing better makes us happier, but less so if those we compare us with experience similar gains (Layard et al., 2010). Even when we do outcompete our neighbor, the *hedonic treadmill* gives us but a temporary peak before our well-being returns closer to its previous baseline (Diener et al., 2009a).

As an alternative to economic pursuits—which often drive non-sustainable growth (Hickel and Kallis, 2020)—well-being scholars have substantiated how other important sources of well-being include good social relations and family life, strong health, firmly held belief systems, and living up to cultural ideals (Nesse, 2005; Haidt, 2007; Diener and Seligman, 2009; Baumeister et al., 2013). Well-being is not only a desirable outcome in and of itself, but mediates important psychological variables. Ill-being correlates with, for instance, extremism (Costabile et al., 2020), anti-sociality (Diener, 2009), and materialism (Hellevik, 2014). The field has established correlations between life factors and well-being, but what well-being should entail, beyond covering basic needs, has engendered a confounding conceptual plurality. Scholars highlight a variety of features of well-being, ranging from hedonistic (pleasure), eudaimonic (self-realization), cognitive (satisfaction), objective (lists of goods), *et cetera* (Røysamb and Nes, 2016). Disagreement on strategy is also considerable. Should we really strive to maximize positive and minimize negative affects (Gruber et al., 2011)? How must we reconceptualize well-being to avoid competition-centered rationality and cultural biases?

Democracy and individual rights have been assumed to explain much of why Westerners report relatively high levels of happiness. Diener et al. (1995) concluded that individualism is strongly predictive of well-being, which has obvious policy implications. Some non-Western thinkers question whether well-being must be assessed at the individual level (Uchida et al., 2009; Uchida and Kitayama, 2009; Rappleye et al., 2020; Krys et al., 2021a,b). Confucianism stresses interdependent well-being—good relationships and social harmony—with which Western concepts of happiness only partially overlap.^2^ In these cultures, well-being should perhaps be assessed at a group level. Diener et al. (2009b) posit that well-being measures are “inherently democratic,” but their concept of “democratic” derives from a distinct tradition (Henrich, 2020). Individuals and cultures can also be averse to happiness. Some Buddhist traditions view a desire for happiness as misguided, some Muslim schools of thought associate happiness with shallowness, and some Russian cultural beliefs point to happiness as often deriving from immoral actions (Joshanloo and Weijers, 2014). These perceptions present a stark contrast to Western views of individual happiness being the basic building block that one can use to justify other values (Braithwaite and Law, 1985).

Vittersø (n.d.) suggests that we can craft a more objective model for well-being by grounding it in the humanistic values of human rights. These rights have not escaped critical scrutiny.^3^ The West may have convinced the UN to declare such rights universal in 1948, but the past decades have demonstrated that not all the world’s peoples want to forge liberal democracies united in a Kantian federation (Larsen, 2022). The 1990s’ end-of-history hubris has given way for a realization of the UN’s Human Rights not being part of a natural law, but rather “the outlines of the common good” as understood from a predominantly Western perspective (Finnis, 2011). Liberal humanism is similarly tied to the region within which it evolved; its concept of “betterment” offers no universal foundation for human aspirations (Henrich, 2020). In developing his humanistic model, Vittersø points to another possible foundation for a culturally sensitive approach to human flourishing: the cooperative universals of our shared nature.

Buss (2000) has offered an evolutionary perspective on why modern ideals and practices do not necessarily make humans happy: environmental mismatch, larger comparison groups, and the benefit of feeling distress when our adaptive strategies fail. Nesse (2005) has elaborated on the adaptive functions of positive and negative emotions. From an evolutionary perspective, happiness is not an affect meant to be maximized, but a semi-transient reward for solving adaptively relevant problems. Many achievements—like money, status, social and professional success—enhance our ability to survive and reproduce. When we progress toward these goals, positive emotions tell us to keep on going (Carver and Scheier, 1990). Happiness is a compass that steers us toward successes that exceed those of our comparison group. The adaptive benefit of sociality (Lewis et al., 2015), as well as indirect fitness concerns,^4^ allow us reap happiness from spending time with our immediate circles and also appreciating their successes.

Such a perspective, suggested Nesse, would offer a theoretical bridge from which we can better understand human emotions as they relate to goal pursuit, as well as suggest policy that aligns with our desires and predispositions. Hill and Buss (2008) noted that with the field’s high stakes, it was “surprising that few researchers have yet to explore subjective well-being from an evolutionary perspective.” Little has since been added.^5^ This article responds to these calls by synthesizing insights from the well-being field under an umbrella of multilevel selection (MLS).

Buss and Nesse’s works on happiness elucidate how certain affects compel people to do well in individual selection. Yet considerable well-being is derived from altruistic behavior that does not directly enhance fitness for the altruist (Thoits and Hewitt, 2001; Musick and Wilson, 2003; Piliavin, 2003; Dolan et al., 2008; Meier and Stutzer, 2008). Baumeister’s (2005) and Baumeister et al. (2013) work on meaning explains how certain affects have evolved to reward people for contributing to their community.^6^ For purposes of analysis and policy recommendation, it can be beneficial to conceptualize well-being as consisting of those clusters of affects that promote, respectively, individual and group selection. We call these clusters *happiness* and *meaning*.

**Figure 1:**
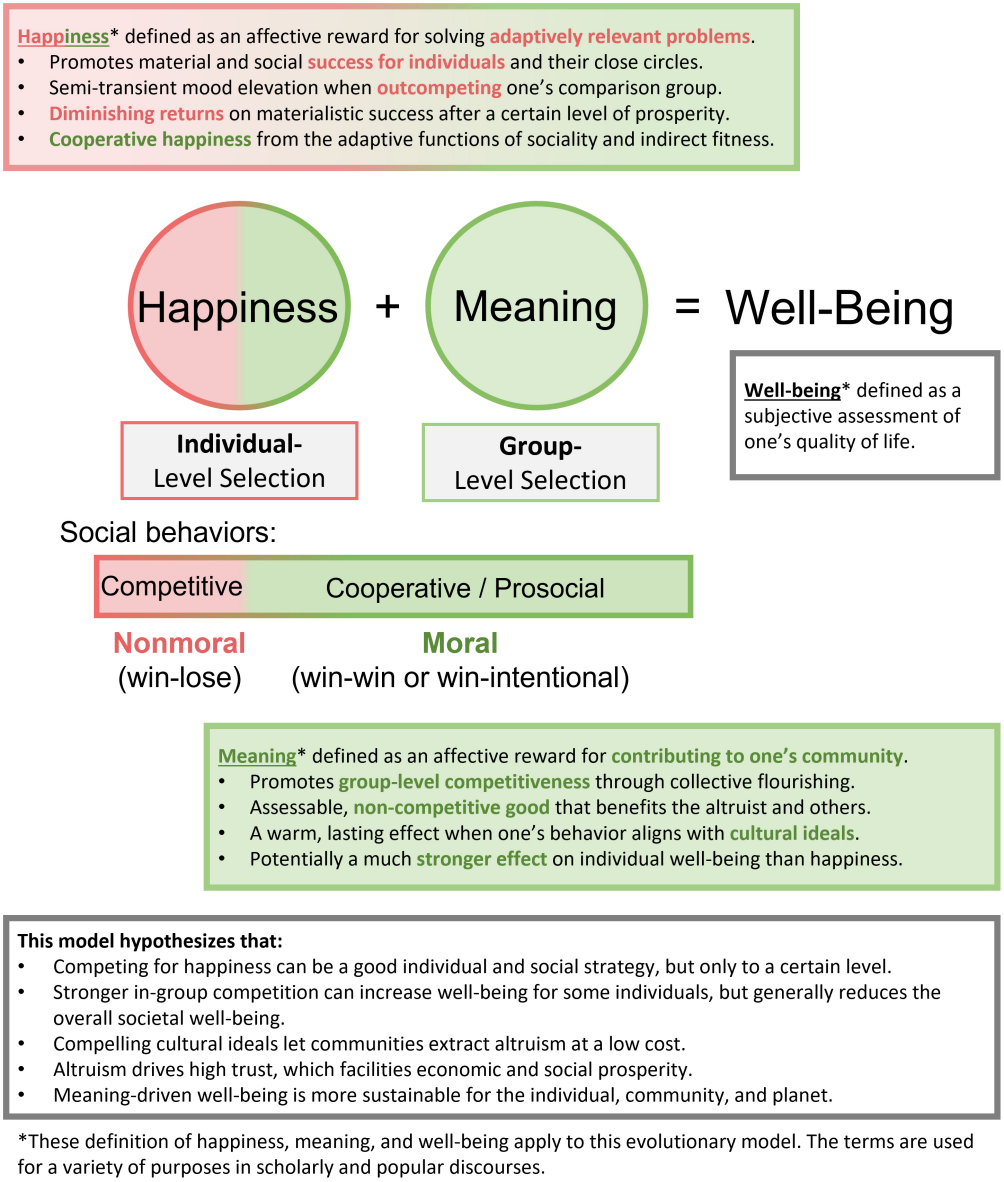
This model attempts to ameliorate the well-being field’s Western-centrism and concept overload while bringing attention to the prosocial, more sustainable aspects of good living.

Since those terms are used in popular and scholarly discourses for a variety of purposes—and vary across language—abbreviating them to “H” and “M” would be an option, yet we prefer familiar words for ease of communication. Approaching well-being in this way lets us bring attention to how public policy, if it is to raise a population’s overall flourishing, should facilitate a combination of working for one’s own success and that of one’s community. Such a strategy appears to contribute significantly to why the Nordics are ranked by the UNDP to be the best countries to live in,^7^ and are among the highest scorers in the *World Happiness Report*. Recent studies suggest that the so-called Nordic “well-being societies” are anchored in high material welfare, a low Gini coefficient, and the highest levels of voluntarism in the world (Witoszek and Midttun, 2018). This model allows more people to solve adaptive relevant challenges and to pursue meaning through state-funded altruistic activities. Figure 1 offers a visual presentation of this model that highlights the prosocial sources of well-being, those that are non-competitive and generally more sustainable.

## 2. Theoretical framework

Our MLS model brings attention to the distinction between the two forms of social behavior: competitive and cooperative/prosocial.^8^ For individuals, engaging in intense in-group competition can be an effective strategy, given that they win. Since such contests produce more losers than winners, increased competition tends to reduce societal well-being (Luthar, 2003). A policy implication could be to enhance opportunity for well-being that derives from meaning and cooperative happiness pursuits such as cultivating high-quality relationships (Lansford, 2000). Especially meaning has the potential to promote individual flourishing in a manner that increases well-being for other community members.^9^

This MLS approach aligns with that of Wilson and Coan (2021). They propose that therapy should be informed by evolutionary insights into the importance of social relations. Viewing individuals as part of social organisms has implications for “improving well-being at all scales, from individuals to the planet.” Their study is a part of Wilson’s advocacy for letting Darwin’s insights inform not only scholarly pursuits, but all forms of governance (Wilson, 2019). We contend that the field of positive psychology, too, could benefit from a keener focus on communities as social organisms with significant influence on individual well-being. The same goes for policy makers.

Our evolutionary past has coded affects into us that help us navigate between personal and communal needs. To understand how this system motivates adaptation on individual and group levels, we need not philosophize in regard to what well-being should be or which affects—or virtues—it consists of. Nature and culture have already negotiated this content within each individual, group, and moral community—with varying degrees of functionality. Understanding how a culture motivates certain behaviors in terms of meaning and happiness calls for deep insights into a community’s history. But to comprehend well-being itself, we need not opine on ontology and semantics, an activity that inevitably is culturally biased. Suffice it to view well-being as a biocultural phenomenon that makes people “feel good” in a manner that motivates them to continue the behavior that triggered this affect.

### 2.1. Well-being

In addition to cultural preferences for well-being, heritability plays a large role. Røysamb and Nes’s (2018) meta-analysis finds that the genetic influence is around 40%. Much of this variability is mediated through personality genes (Weiss et al., 2008; Keyes et al., 2015). That some individuals and groups have a lower baseline matters less for evolutionary functionality. The fitness value of these emotions depends on how they play out in those situations in which they are adaptive. It is not a person’s baseline of well-being that provides the primary signal, but to what extent their well-being increases or decreases in response to a particular circumstance (Nesse, 2005).

Those mechanisms align with individual and group needs. When something hinders an individual’s access to adaptively relevant resources, their mood response (1) brings their attention to the source of the strategic interference, (2) prompts them to remember this information, (3) motivates them to reduce the interference and (4) prevent future interreference (Buss, 1989; Hill and Buss, 2008). During crisis, individual response appears to have evolved also to meet group needs. Reduced happiness seems to trigger a desire for meaning as a means for elevating one’s well-being. Early in the corona pandemic, the world experienced a doubling of the proportion of individuals who chose to help strangers—precisely when more people needed help. Donations and volunteering were up too (Helliwell et al., 2022). In times of need, cultural ideals that motivate altruism can provide advantage in terms of group selection. A community in which people take care of each other will have a stronger cohesion than one dominated by selfishness (Wilson and Hessen, 2018). In war, such sacrificial zeal can be decisive (Atran et al., 2014). By selecting to interview volunteers who help refugees from war—one group from the same Slavonic cultural sphere as the refugees, the other from a different culture—we gain insight into how emotions triggered by grave conflict contribute to altruistic motivation.

An evolutionary approach to these emotions brings our attention to the importance of a change in intensity. Someone who always feels good—or bad—would not benefit from the signals that mood changes provide. Neither will such signals always trigger adaptive response. Evolution shaped these affects so that they should, on average, motivate behaviors that tend to maximize reproductive success (Nesse, 2005). Emotions will regularly arise from misunderstandings or motivate dysfunctional response. In the modern world, environmental mismatch further complicates the calculations of these biochemical algorithms (Durkee et al., 2019).

### 2.2. Happiness

When we progress toward solving adaptively relevant problems, the intensity level of the affective reward is influenced by the rate of progression in relation to expectations, and by how members of our comparison group are faring. Modern media that contrast our own status, beauty, wealth, competencies, and performances with vastly larger groups than those of our ancestral environment do not promote happiness (Hill and Buss, 2008). Increasing inequalities contribute to this malaise. Our minds seem to have a *positional bias* that drives us to judge success in resource acquisition not in absolute terms, but compared to our chosen peers (Frank, 1999; Hill and Buss, 2006).

These mechanisms make it more profitable for people—in general—to invest more in happiness’s cooperative sources because these are likelier to pay off. More accessible happiness derives from the adaptive functions of mating, friendship, kinship, and coalition (Buss, 2000). While such sociality is collaborative, it includes an element of competition; cooperative versus competitive distinction involves a scale. Allocating more resources to cooperative pursuits is less incentivized in individualistic, competitive cultures.^10^ Social democratic ideology, which sacralizes work-life balance (Schulz, 2010), contributes to why Nordics rank among the happiest people on earth (Helliwell et al., 2022). Their relative success exemplifies how, while culture influences what provides meaning, it also sets parameters for happiness. An important part of individual success is to perform well in one’s social role (Baumeister, 2005). Societies with high well-being align many paths to happiness with what also serves the community, so that meaning and happiness pursuits contribute to individual as well as societal well-being.

Defining happiness as an MLS phenomenon informs positive psychology’s dispute in regard to which social unit should be primary for analysis (Krys et al., 2021b). Whether independent or interdependent happiness pursuits are more adaptive depends on the environment. In kinship societies, the well-being of the kin group is of such importance to each member’s fitness that interdependent concerns take precedence (Henrich, 2020). In Western societies, individual strategies are more relevant. An MLS perspective also provides a temporal axis. Happiness is largely present oriented, reflecting an individual’s needs and wants. Meaning is future oriented, seeking to integrate an individual’s past and present experience with a collective goal that, the further ahead it lies, the deeper the meaning it can provide (Frankl, 1988; Baumeister, 2005).

### 2.3. Meaning

A quest for meaning is uniquely human, a cultural tool that motivates behavior that benefits the collective. Warneken and Tomasello (2009) place the roots of altruism—in the form of instrumental helping—to our last common ancestor with chimpanzees. Yet chimp prosociality limits itself to close kin, small hunting groups, and boundary enlargement. Other social behaviors tend to be highly competitive; chimps seem not to care much for the well-being of non-kin (Silk et al., 2005; Wrangham, R. W. 2019; Wrangham R. 2019). With the evolution of culture, *Homo sapiens* could extend natural predispositions for nurture to non-kin, and even strangers.

Such prosociality, boosted by cultural norms and ideals, made large-scale sociality possible. Meaning was crucial to this scaling up; it is the very fabric of culture, our ancestors’ only means for storing and imposing complex information on large systems (Baumeister, 2005). Cultural ideals facilitate prosociality by turning what benefits the group into intrinsic motivation for group members. By acting in ways that one’s culture defines as meaningful, individuals are rewarded with increased self-esteem, a crucial component of well-being (Solomon et al., 2000; Kirkpatrick and Navarrete, 2006).

Self-esteem is adaptive because it results from, and reinforces, social belonging (Baumeister, 2005). In Western philosophy, altruism is mostly conceptualized as something that cannot have a selfish component (Ricard, 2015). Classical economics posited that acting in selfish interest will aggregate to communal good, but the act itself was conceptualized as selfish (Smith, 1759). In Christian thought, helping those in need was a virtue, but such altruistic contributions should be offered without concern for one’s own worldly benefit. Building on Darwin, Spencer (1892) separated altruism’s primary and secondary effects, reconceptualizing the relationship between beneficiary and altruist. An evolutionary perspective illuminates how this biocultural mechanism is effective precisely due to its win-win nature. Darwin (1871) considered it to be “the noblest part of our nature,” how *Homo sapiens* can extend our nurturing instinct to larger circles, from offspring and kin to social groups, and nations, and even other species.

## 3. Materials and methods

Positive psychology has relied almost exclusively on quantitative forms of research. The field’s scholars have established a correlation between prosociality and well-being, as well as some of the mechanisms that underpin this correlation. Yet quantitative research falls short of explaining these mechanisms at a deeper level (Hui et al., 2020; Moche and Västfjäll, 2021). Experimenting has been of limited utility in this pursuit (Charness and Grosskopf, 2001; Konow and Earley, 2002). Meier and Stutzer (2008) explain the suboptimal outcome of laboratory studies with how such stimuli provide too low-stakes to influence reported life satisfaction.^11^ A line of field studies with longitudinal survey data has confirmed positive effects from volunteering (Wilson and Musick, 1999; Thoits and Hewitt, 2001). To investigate how and why altruistic behavior contributes to the altruist’s own quality of life, we first conducted a quantitative study to confirm the correlation between prosociality and well-being at a national level. Following up with in-depth qualitative interviews allowed us to examine the deeper mechanisms that contribute to this correlation.

### 3.1. Preliminary quantitative study

We obtained national-level indicators of prosociality and happiness from several public, cross-national databases. For markers of prosociality, we adopted the trust scores from World Value Survey (Waves 6 and 7, Inglehart et al., 2014; Haerpfer et al., 2020) and cooperation scores from a cross-cultural experimental study (Romano et al., 2021). For markers of happiness, we use Life Ladder, Positive and Negative Affect from the *World Happiness Report* (Helliwell et al., 2019). See Table 1 for brief descriptions and hypothetical score ranges with labels.

**Table 1:** Descriptions and zero-order correlations of the national focal variables.

**Variables**	**Descriptions**		**1**	**2**	**3**	**4**	**5**
1. Trust (WVSw7)	The percentage of people indicates “Most people can be trusted” (versus “Need to be very careful”), 0–100%.	*r*	—				
		*p*	—				
		*k*	—				
2. Trust (WVSw6)	The national aggregation of the average scores of “how much you trust people from various groups,” e.g., people you know personally, 1 = Trust completely; 4 = Do not trust at all.	*r*	−0.90***	—			
		*p*	< 0.001	—			
		*k*	34	—			
3. Cooperation	The national aggregation of the one-shot decisions in a prisoner’s dilemma game in an online experiment. Participants were asked to allocate up to 10 monetary units to their partners, 0 = none; 10 = all.	*r*	0.13	0.13	—		
		*p*	0.51	0.58	—		
		*k*	28	21	—		
4. Life Ladder	National subjective well-being “On which step of the ladder would you say you personally feel you stand at this time?” 0 = the worst possible life; 10 = the best possible life.	*r*	0.59***	−0.49**	0.53*	—	
		*p*	< 0.001	0.01	0.02	—	
		*k*	38	31	20	—	
5. Positive Affect	National average scores of positive affect measures from *World Happiness Report*, e.g., “Did you smile or laugh a lot yesterday?,” 0–1	*r*	0.28†	−0.32†	−0.05	0.49**	—
		*p*	0.09	0.08	0.84	0.001	—
		*k*	38	31	20	42	—
6. Negative Affect	National average scores of negative affect measures from *World Happiness Report*. “Did you experience the following feelings a lot yesterday?,” e.g., Worry. 0–1	*r*	−0.47**	0.44*	0.12	−0.38*	−0.41**
		*p*	0.001	0.01	0.62	0.01	0.01
		*k*	38	31	20	42	42

***p < 0.001, **p < 0.01, *p < 0.05, †*p* < 0.10; *r*, Pearson’s *r*; *k*, number of nations with available data. WVSw7, World Value Survey wave 7; WVSw6, World Value Survey wave 6.

Table 1 shows that there were significant correlations between the indicators of prosociality and those of well-being at a national level of analysis. Trust and Cooperation were strongly associated with higher subjective well-being (|*r*|s > 0.49, *p*s < 0.02). Trust was strongly associated with lower negative affect (|*r*|s > 0.44, *p*s < 0.01) and moderately associated with greater positive affect at a marginal significance level (|*r*|s > 0.28, *p*s < 0.09).

### 3.2. Qualitative interviews

Nesse (2005) believes that such quantitative approaches mostly provide shallow insights. He is critical to how positive psychology has been so preoccupied with establishing correlations between external circumstance and subjective well-being. If human flourishing is not best understood as a consequence of life factors, but of the ways in which individuals interpret and adapt to evolved signals, “survey studies of well-being will overlook most of what is important.” Nesse concludes that “implications for methodology are severe [as] only narrative includes information detailed and idiographic enough to allow a real understanding of an individual’s life.”

#### 3.2.1. Study population and design

The methodology of our main study aligns with Nesse’s emphasis on a qualitative approach. To investigate and problematize the meaning part of our equation, we conducted in-depth interviews with 32 informants. We recruited dedicated altruists to access thick descriptions of prosocial motivation. Most informants were recruited *via* local chapters of the Red Cross. We recruited a few informants who had appeared in news media as helpers of Ukrainian refugees or been active in social media forums dedicated to such activities. We do not assume that all personality types gain similar rewards from altruistic behavior. Studies show that extrinsically motivated materialists benefit less from volunteering than people with intrinsic life goals (Meier and Stutzer, 2008). Our priority was through our purposive sampling to gain info-rich access to the narratives of people who had considerable experience with, and reflection around, altruistic work.

We recruited 16 women and 16 men aged 23–80. Opting for a broad age range allowed us to investigate how people’s views and practices with regard to altruism vary across life stages. Nearly all informants were long-term residents of Norway. Fourteen identified primarily as Norwegian, 1 as Swedish, 10 as Polish, 4 as Ukrainian, 1 as Russian, 1 as Belarusian, and 1 as Latvian. Three scholars conducted semi-structured interviews in Polish, English, Norwegian, and Norwegian-Swedish. We began by asking open-ended questions about which activities the informant was engaged in. We would later focus on emotional experience during and after altruistic practice, cultural influences, and long-term individual and social effects. Interviews in Polish were transcribed manually—the other ones *via* software, then quality-proofed manually. Direct quotes have been edited for readability. We use this research material in another article that investigates cultural modes of altruism. To preserve informant privacy, interviews are not made available.

Our grounded theory approach entailed an interplay between data collection and analysis throughout the interview period March–June, 2022. Twenty interviews were in person, while 12 were *via* Zoom due to those informants’ remote location. All respondents gave informed written or oral recorded consent. Since we have a relatively large sample size for a qualitative study of this type (Marshall et al., 2013; Schreier, 2018), we choose not to name informants, but to use nationality, gender, and age. We respected the request of female informants of Polish extraction who preferred not to disclose their age and proposed that we instead use their first names. We stopped recruiting informants when reaching saturation in terms of novel information per interview. Ethics approval was obtained in line with the Norwegian decentralized model. Our project was assessed by the Norwegian Agency for Shared Services in Education and Research (reference number 445357).

#### 3.2.2. Cultural comparison

We recruited informants with different cultural backgrounds to investigate how modes of altruism influence meaning-making. The Nordic region has centuries of positive experience with prosocial collaboration across social spheres (Witoszek and Midttun, 2018; Hänninen et al., 2019). Choosing Lutheranism as their Protestant creed set Nordics on a path different from those of other Europeans (Fukuyama, 2014). An egalitarian, prosocial ethos made it everyone’s responsibility to ensure everyone else’s well-being, as from pauper to king, all were meant to be united in a “priesthood of believers.” Social democracy can thus be understood as a secularized version of Lutheranism that offers modern justification for enduring practices (Witoszek, 2012; Nelson, 2017).

The Slavonic region^12^ was less influenced by the practices that drove WEIRD^13^ psychology (Henrich et al., 2010; Henrich, 2020). A history of centralized, authoritarian rule and their recent communist past cast long shadows of distrust (Meier and Stutzer, 2008). Among Norwegians and Swedes, 72 and 63%, respectively, think “most people can be trusted.” 23% of Russians, 24% of Poles, 30% of Ukrainians, and 40% of Belarusians think the same (Haerpfer et al., 2020). Living in the former Soviet Bloc, which by many was experienced as a deeply decivilizing process, is a strong predictor for negative affect (Deaton, 2010). While Nordics are exceptionally trusting of their governments, the dominant attitudes to institutions in post-communist countries are mistrust and subversion. 67% of Norwegians report doing organized altruism. Poland, Russia, and Ukraine’s percentages are in the mid-10s to low 20s (Huppert et al., 2009). Informal volunteering distributes similarly (Plagnol and Huppert, 2010).

Our analytical approach aligns with what Wilson and Coan (2021) refer to as uncovering the assumptions that culture makes invisible. We performed a semiotic analysis of our interviews, investigating how cultural narratives, tropes, and symbols inform individual experience and meaning-making (Eco, 1986; Lotman, 1990). Our qualitative study is evocative of the constructivist-interpretive tradition (Levitt et al., 2017), although our evolutionary perspective provides a meta-narrative with different implications than the constructivist one. We analyzed interviewee responses to illuminate universal predispositions for altruism; and importantly, how these are strongly mediated by culture. An important focus was how distinct cultures have different capacities for motivating prosocial contributions.

## 4. Results and discussion

The Russian invasion of Ukraine triggered strong emotions for many of our informants. The Slavonic volunteers reported being particularly distressed as the war felt more personal due to historical experience of Russian and Soviet oppression. The resulting ill-being motivated them to seek activities that could provide socialization while helping victims of war. The majority had no experience with voluntarism. Nearly all overworked themselves, often to the detriment of family, work, education, and/or other individual concerns.

Their voluntary engagement was marked by a powerful emotional response to the invasion of Ukraine and their strong identification with the Ukrainian refugees. Many Polish volunteers felt that the Ukrainian war was also their war, so that they had a particular responsibility to contribute. This stronger emotional investment infused their lives with meaning, guiding their actions during a difficult time and helping them set inspiring goals for the future. Many informants felt empowered, expanded their social circles, experienced personal growth, and became eager to advocate the benefits of altruism. A Ukrainian female (32) said, “What is happening to me now is like a whole new life. I was someone who could not make any decisions on their own or fix things. I needed help. But not anymore. I feel like I’m bigger on the inside, that I have room for more people.”

The Nordic helpers were more motivated by living up to cultural ideals of altruistic universality. Most Norwegians had years of experience with voluntary work. They were socialized into the Nordic way of helping, marked by lower emotional intensity, trust in state-supported humanitarian structures, and prioritizing long-term efficacy over short-term emotional satisfaction. Many felt that Nordics have a particular duty to help because their societies are so rich and well-functioning (Oxfeldt, 2018). In spite of these cultural differences, the narratives of all our informants spoke of the importance of meaning for their quality of life—especially during difficult times—and some attested to the group selection function of this cluster of affects.

### 4.1. Help others to help yourself

Our informants experienced the most intense affect when helping refugees face-to-face. “Putting the soup bowl directly in the hand of the refugee is what feels best,” explained a Norwegian (54), “that’s almost euphoric.” He quipped that “Mother Theresa was the world’s biggest egoist, because she got the best feeling.” His experience as a volunteer near war zones had given him insight into altruism with the highest emotional potency. He had worked with helpers who were so intoxicated by meaning that they neglected their own physical well-being. He suggested heroin as an analogy to how some volunteers crave a sustained super-intensity of emotion. Many disregarded professional work, personal economy, and social obligation to structure their life around contributing to those most in need. Our informant decided to forego this intense emotional reward, instead dedicating his voluntarism to providing logistics for volunteers so that they would suffer less and “as many as possible can have that feeling.” Polish Anita made a similar analogy: “I was on a total high, as if after taking drugs. You know you are reaching your limit, but you also know that the stress will not break you. That’s a great feeling.”

Experiencing altruistic reward, several Slavonic informants bemoaned how volunteerism had been disincentivized and atrophied in their native countries. “I wanted to help the homeless,” explained a Ukrainian female (38), “They get no help from the state, but I felt so removed from them that I was scared to help.” A Ukrainian male (37) said, “Working together is the only way to build trust. For now, in Russia and Ukraine, people generally do not trust government, police, doctors—it’s a sad life.” A Belarusian male (42) said, “We fear scams, spies, propaganda. We want to help, but we have to focus on our own house. I cannot tell my friends in Belarus that I do voluntary work now. They would think I was a political activist only to please the party to earn money for myself.” A Norwegian (47) met distrust from the Ukrainians she helped, “They are not used to a culture of acting selflessly. They have asked us, ‘Why do you do it? What’s in it for you?’” Polish Ewa explained that the Poles “are still haunted by the post-communist legacy which is about zero trust in the state and its institutions. We prefer to act outside the system, we feel safer in relating to a person more than to an institution.”

Centuries of close, effective cooperation have created virtuous circles in Norwegian communities. Narratives of prosociality—taught in schools, dominant in family stories, and replicated by cultural heroes—are so ingrained in the social fabric that volunteerism comes at a low cost. Western philosophy’s emphasis on *unselfish* altruism created dissonance for several informants. “I cannot distinguish whether I do this to help others or myself,” said a Norwegian (33). To downplay his own virtue, he would tell friends that his motivation was purely selfish.

Another Norwegian (23), who structured his life around volunteerism, conceded that “being part of a fellowship is primary, helping is a bonus.” Interpersonal altruism feels good, but a sense of mastery and “being part of a gang” are similarly important. Seeing immediate results in your local community is most rewarding. Positive feedback makes him feel seen as a person of value, which is “extremely important.” Quantitative studies confirm the importance of social affirmation in motivating prosociality (Harbaugh, 1998). Our informant noted that volunteers “are often not the coolest guys in class.” For them, voluntary organizations provided alternative arenas for personal growth. These dynamics illuminate why many voluntary organizations have a preponderance of retired members. Several informants bemoaned how difficult it was to recruit young people to altruistic activities, but were also understanding. Most of these seniors had only dedicated significant time to helping others after they retired. Losing access to professional pursuits that could generate happiness through individual success, as well as meaning through contributing to others, motivated pensioners to engage in voluntarism. Young people striving to establish a career, a family, and social network face a different equation for well-being. The Norwegian 23-year-old had a background of health and learning challenges that had motivated his voluntary engagement. How he referred to other young participants as often not being “the coolest guys in class” attests to how meaning-generating activities can be a more readily available source of well-being. Such activities offer belonging and a sense of communal contribution and mastery. Our informant told us that each time he had succeeded in arranging events with an altruistic purpose, he enjoyed an affect that made him look forward to succeeding with future such events.

A Norwegian male (60) confirmed how structuring altruism around rewarding socialization heightens motivation. Another Norwegian (80) gained self-confidence after accepting a local leadership position in the Red Cross. He felt heightened self-esteem, people viewed him differently, and he was invited to speak on the National Day. This gave a good feeling, greater self-respect, and joy. Volunteers valued these direct benefits, but seemed to derive greater reward from contributing to the social and professional success of refugees. “It feels so good to see them succeed in life,” said a Norwegian male (68). “Seeing that what I do helps makes me feel better,” said a Ukrainian (38), “I get back double what I give.” Polish Agata said, “I met gratitude, people telling me that I was an angel, and that was fantastic, although sometimes embarrassing. It created meaning.”

### 4.2. Transient happiness, sustainable meaning

Compared to the reward our informants experienced from individual success, contributing to the well-being of others produced affect that has been more enduring. Several informants used the imagery of building blocks to describe how they experienced success in competitive pursuits. When our Belarusian informant (42) earned a PhD, this produced a happiness peak, but the next day, he felt about the same as he had before. He and other informants spoke of professional success as something that mostly lets us move on to our next goal. When he told stories of his altruism, these memories rekindled the warm affect his actions had inspired. Informants felt good for weeks after helping, some believing these experiences could contribute to their well-being for a lifetime. Happiness is fleeting, many concluded, while meaning sustains them in the long run.

**Figure 2:**
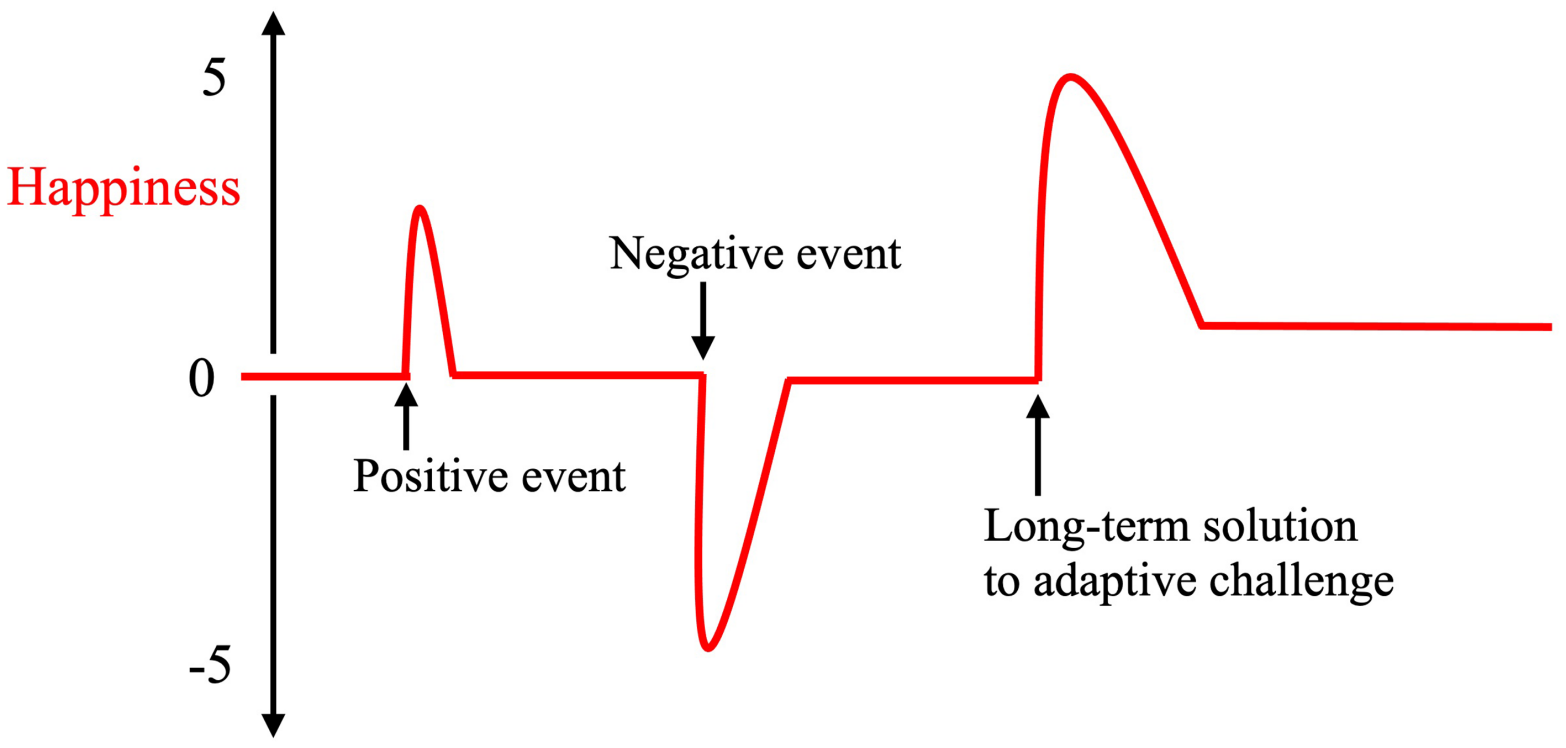
Experiencing events that trigger positive affect does not make one’s happiness increase in an accumulative manner. When individuals forge sustainable solutions to adaptive problems, their happiness level can increase. Consequential failure can lower one’s baseline. Adjusting one’s goals and expectations to better fit opportunity and talents can also have a positive effect.

The hedonic treadmill of happiness has confounded scholars (Diener et al., 2009a). If we view this cluster of affects as an evolutionary signal, a too strong accumulation of happiness would reduce signal sensitivity. Such a perspective offers a different take on *prospect theory*, as well (Kahneman and Tversky, 1979). This theory’s S-shaped curve in terms of how people value gains and losses must not necessarily be interpreted as diminishing *utility* along the axis’s concavity. Our appreciation for consecutive gains could diminish to maintain signal sensitivity for the neurocognitive suite that governs our well-being. Figure 2 illustrates how succeeding with adaptively relevant problems rewards individuals with a happiness peak before well-being returns closer to their baseline. Instead of conceptualizing happiness as increasing on a scale from 1 to 10, we use a baseline of 0 with 5 steps in each direction.

Research shows that after individual success, one’s baseline can end up elevated (Diener et al., 2009a). As adaptive challenges are solved, an individual can perhaps afford reduced signal sensitivity in the positive part of the scale. Expectations too play in. Nesse (2005) argues that an increase in sustained well-being results from having interpreted environmental signals adaptively and adjusted one’s life goals accordingly. When people have enough time, energy, and resources successfully to pursue all they experience to be important, they should enjoy high well-being. They are also more likely to be pro-peace and cooperative in their attitudes (Diener, 2009).

Similar dynamics play out in societies like the Nordic ones. Having implemented more effective solutions to modern challenges, communal well-being has increased in spite of the Easterlin paradox. The Nordic people are among the world’s most prosperous, but their social democratic model facilitates that they are also among the developed world’s most income-equal (World Bank, 2019; CIA, 2021). Tellingly, Danes are happier than Americans primarily because their poorest do better (Biswas-Diener et al., 2010). Given the diminishing returns on materialistic success (Piketty, 2022)—and the negative effect of envy—spreading economic resources makes sense from a perspective of societal well-being (Diener et al., 2009c; Biglan et al., 2020). Facilitating practices that let the better-offs derive meaning from contributing to those in need is another way to exploit our shared nature to increase overall well-being. A Ukrainian female (41) suspected that such dynamics partially explain Nordic generosity, “They engage abroad and with refugees to get a contrast to their own perfect lives.” Several Nordic informants confirmed that helping refugees was more rewarding due to the contrast between their own well-being and the other’s hardship. A Norwegian informant (60) found it “more meaningful to help refugees get the basics than to help others get more luxuries.”

Good feelings are often important for motivation. According to Baumeister (2005), meaning requires that the altruist has a subjective perception of efficacy, as future-oriented goals without the ability to achieve them will make people feel helpless. An illusion suffices; as long as people feel useful, positive affect follows. A Ukrainian helper (38) said that what you do must feel meaningful, or you cannot volunteer—you “must leave a mark.” A Norwegian (23) concurred, “If you are not making a difference, you lose motivation.” Quantitative studies substantiate the importance of perceived utility (Argyle, 1999). These mechanisms attest to the complex interaction between individual and communal needs, negotiated through environmental signals and interior states. Individuals may not fully understand why they are driven to sacrifice, but in a functional community, such contributions are reinforced through psychological and social rewards.

Meaning-generating activities do not necessarily trigger positive affect in the present. Certain forms of altruism produce peaks of positive affect, but meaning seems, to a greater extent than happiness, to accrete to a lasting sense of well-being. This process can involve a slow recognition of one’s own potential and sometimes a discovery of hidden talents. Many of our Slavonic informants reported short-term ill-being from their altruistic contributions, a common outcome with especially demanding meaning pursuits (Dakin et al., 2021). Strong identification with victims of war was so emotionally draining that the volunteers felt worse after long days at the refugee center. They made this sacrifice because they wanted to alleviate trauma for people with whom they felt affinity. Polish Alexandra said, “Nothing is more meaningful than helping others to restore their dignity and humanity.” In spite of initial desperation, all Polish informants concluded that in the long run their lives became richer and more meaningful. Their experience testifies to how well-being is not necessarily identical to feeling “good.” Individuals with low happiness level sometimes refer to such meaning-driven well-being as feeling “right.”

For many of our informants, prosocial contributions could generate high well-being for weeks or months. Still, for meaning to contribute to a relatively high level of well-being, the altruistic activity had to be ongoing. Several informants longed back to voluntarism if they had been inactive for too long. Helping new people elevated their sense of meaning, and thus well-being, to previous levels. Quantitative studies support that well-being diminishes when people stop volunteering (Meier and Stutzer, 2008). A Polish male (38) said, “In the future, I want to shift from being a software expert to establishing my own charity.”

The diversity of experience among our Nordic and Slavonic informants invites a conceptualization of our well-being system as consisting of two accounts for meaning: one anchored in present-day activity and one that accretes along a lifetime. Having previously invested much in prosociality provided a sense of meaning that did not diminish with time, but which provided less intense affect than ongoing voluntarism. To get out of a rut, informants had to invest in present-day altruism, which required sustained efforts. They did not seem to experience diminishing returns on altruism, an observation supported by studies that establish a correlation between well-being and volunteering frequency (Meier and Stutzer, 2008). Neither did maintaining a certain level of meaning seem to require increased efforts.

There is an additional consideration. Engaging in meaningful activities does not always coincide with a heightened well-being in the short run. A Norwegian female (75) seldom felt happy when helping refugees. She was often tempted to prioritize friends, but doing what she had committed to elevated her quality of life in the long run, “I always feel that I have meaning, but I sometimes lack happiness. I may need to search a little, but meaning is always there. Occasional happiness is a bonus.” Polish Ewa said, “You see too much tragedy. You are often frustrated, feel like climbing the walls. But since I started working at the refugee center, I sleep better.” A Ukrainian volunteer (41) felt terrible when the war broke out, but after helping refugees, “my depression was gone.”

**Figure 3:**
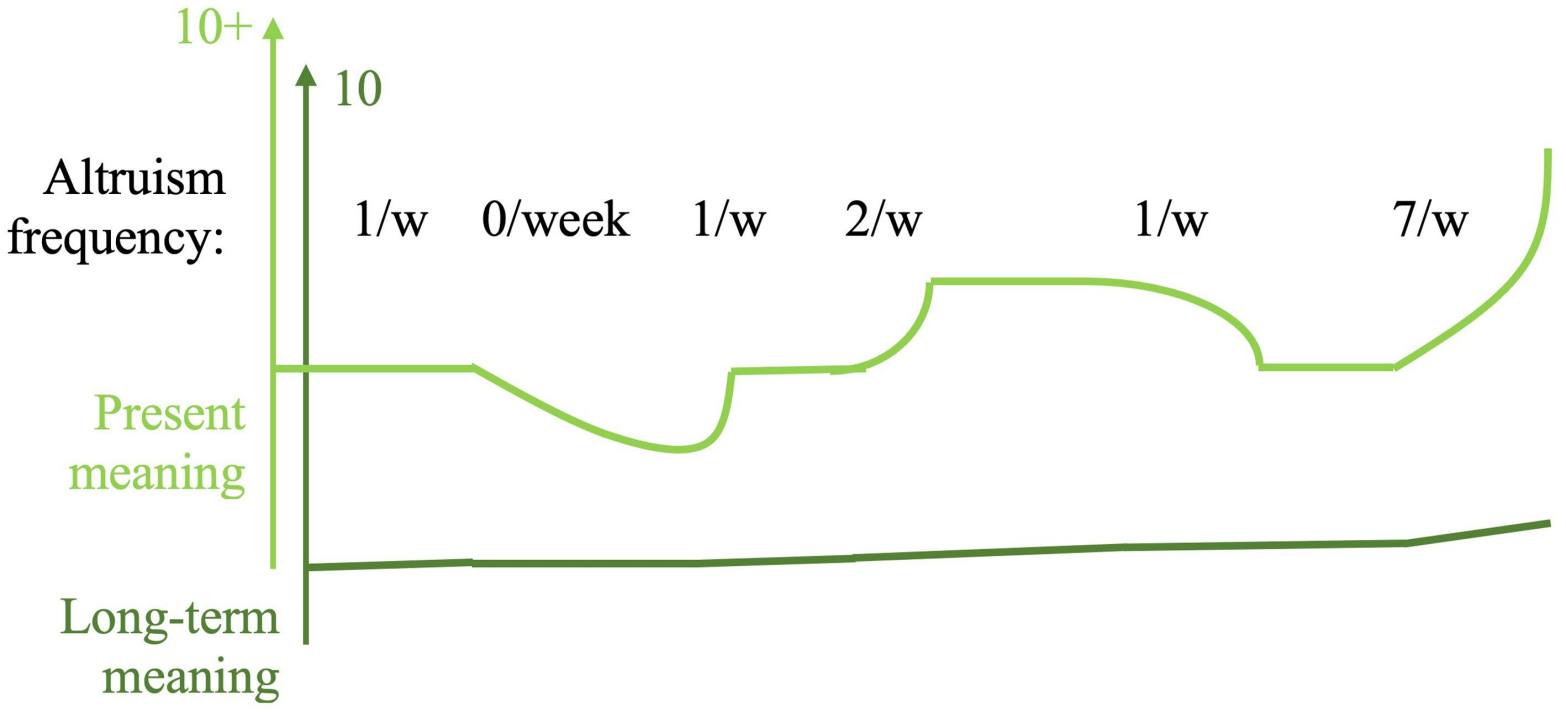
We conceptualize meaning to consist of two accounts. The long-term one fills up as a consequence of altruistic contributions across a lifetime, but provides affect with lower intensity. The present account responds to ongoing altruistic activity and can in extreme cases provide exceptional levels of well-being. Altruists who strongly identify with traumatized victims may experience short-term ill-being.

Quantitative research confirms that helping makes people feel better. That this affect motivates voluntarism is supported by a study that revealed how when people have been convinced that altruism will not elevate their mood, they are less likely to help (Cialdini et al., 1973; Manucia et al., 1984; Wilson and Musick, 1999). Our interviews indicate a need of a more nuanced approach to these mechanisms. In spite of frustrations, many informants continued to help refugees not because they expected immediate mood elevation, but long-term spiritual and moral rewards. Figure 3 illustrates how we conceptualize altruism to generate a sense of meaning on a scale from 1 to 10—or in extreme cases, much higher. Since this affect assesses social belonging (Baumeister, 2005)—which is fixed in relation to a community—it needs not return to a baseline to maintain signal sensitivity. Instead, a group member’s short and long-term meaning accounts are filled up as payment for altruistic contribution in the present and across time. The more you live up to communal ideals, the “better”—or more “right”— you should feel. These dynamics made several of our informants use altruism as a way out of depression.

### 4.3. Meaningful activity as a first step toward well-being

For people with a low level of well-being, it can be hard to find motivation to compete on happiness markets. A Norwegian informant (33) knew from previous depressions that voluntarism is an easily accessible way to feeling better. During his school days, the members of his in-group had derived well-being from supporting each other’s high professional ambitions, “but reality catches up with you.” To volunteer, all you need is to contact a local organization. After entering the job market, he became depressed for not having a position that matched his education. Feeling that life passed him by, he put on 20 kilos before voluntarism helped elevate his mood. Improved well-being motivated him to earn a job he is happy with. Feeling bad during the pandemic, he volunteered at test centers, “so that I could feel useful for society. Being part of the solution gives self-confidence.” Scrolling news from Ukraine, he again “felt meaningless.” Volunteering, now at a refugee center, elevated his mood: “It is a good way to get started with something.”

### 4.4. Meaning as a group selection tool

From our MLS perspective, if meaning-producing mechanisms are functional, they should enhance a community’s chances in group selection. So far, large-scale immigration has not undermined the cultural factors that underpin social democracy in Norway. Against many predictions, social trust reached a new high in 2019 (Haerpfer et al., 2020). Several informants chose to help refugees to strengthen the national community. A Norwegian male (66) said, “I feel a strong urge to support the Nordic model, to live up to its ideals. It is innate to humans to help, which aligns with our political model. Norwegian culture gives power to the experience of helping, so this sense of meaning that I get can be very intrusive. Given that the refugees are here, it is in our self-interest to integrate them so that they end up on the good side, as constructive, tax-paying members of society, not welfare recipients.”

“Generally, integration of immigrants has not been good,” said a Norwegian male (67), “so it is meaningful to help refugees succeed. If more people helped immigrants, they would see that they are not threats. This could reduce polarization.” A Norwegian helper (75) wanted to preserve equality, an important Nordic value: “By helping refugees succeed, economic differences can be kept at an acceptable level.” A Norwegian female (63) wanted to speed up integration, “I use of my own extra energy to show refugees how Norwegian culture and systems work.” From the perspective of our genes, opening one’s borders for different ethnicities is not a strategy with obvious utility. Baumeister (2005) believes that other concerns have a stronger influence since we “identify ourselves with our cultural identity as much as with our genetic makeup, and perhaps more.” Welcoming in and passing Nordic values along to immigrants could be viewed as a way to promote the long-term viability of a community whose fertility rate has dropped far below replacement level (SSB, 2021).

Many Slavonic informants too were driven to strengthen the national community to which they now belonged. A Ukrainian (32) said, “I have always loved life since I got here. It is completely the right country for me, and I always say I was born to live in Norway.” Another Ukrainian (41) wanted to function as a bridge since “there are big cultural differences, and I know both systems. I want to help Norway not by creating anything new, but transporting meaning between peoples who cannot understand each other, so we can move forward together. I can help this go faster. If Ukrainians can think like Norwegians, they can participate in society instead of burdening tax payers.” A Russian female (38) said, “The way we help refugees, we are a bridge between what Norwegian organizations offer and what refugees need right away.” A Norwegian female (37) also used the bridge metaphor, adding, “I want to help open Norwegians more up to those who are different, because we must see people as people, there is no other way.”

Not all of the Slavonic informants expressed a primary allegiance to the Norwegian culture. In spite of being a long-term resident of Norway, a Ukrainian helper (41) did not feel a sense of belonging to her new community: “When I volunteered for refugees before, it was for work experience, it felt like a job.” Using voluntarism to enhance one’s chances on the labor market is a common extrinsic motivation (Menchik and Weisbrod, 1987; Hackl et al., 2004). “Now that I can help my own people, I feel so much better,” said our informant who had suffered guilt induced by having emigrated: “I used to feel that I was getting too much out of life.” The war had made her feel terrible, but now, “When I am with my own at the refugee center, I feel calm, I get my quality of life back, it reduces my anxiety. Improving the lives of people I feel tied to is so much more rewarding.” The Belarusian (42) wanted his voluntarism to benefit his new and former community. Slavonic nations should copy Nordic practices to receive similar advantages, he suggested, “Norwegians are very naïve, thinking everyone is honest like them. But we must choose to be optimistic about the future, we must risk to be naïve. We are all in the same boat. A good future requires that we cooperate. I have a 100-year perspective on my helping. I want a better world for my children.”

## 5. Conclusion

This investigation of the Ukrainian refugee crisis points to how meaning-making activities can benefit individuals and communities. Emergency situations, such as war or refugee influxes, can have a transformative role in terms of promoting prosociality at several levels. On a personal level, helpers can develop a more inclusive, hospitable self, acquiring an expanded identity of caring creatives. On a cultural level, they get training in dialogic imagination, becoming better communicators, listeners, and cooperators. On an evolutionary level, helpers develop skills they did not have before, stretch their potential for adaptation in situations of stress, and increase their resilience. On a political level, in a democratic state, their work can lead to institutions correcting themselves, becoming not just more efficient, but also more humane.

This investigation also attests to the utility of our MLS model for prosocial well-being. We are individuals doomed to strive in status contests, but our communities are also dependent on our altruistic contributions. Our well-being system helps us navigate these pressures. A key to Nordic success is aligning what benefits the individual with what strengthens the community. Several informants praised how Nordic egalitarianism, in combination with jobs that give a sense of communal contribution, had been the foundation for their high quality of life. When success at work feels like altruism, this has a compounding effect. For those without such work, and many retirees, voluntarism filled up their meaning accounts in a similar manner. Since the happiness-side of the equation is inherently relative, we propose that public policy aimed at enhancing meaning has the greatest potential for increasing societal well-being—especially in developed countries.

This conclusion is supported by studies that attest to an intriguing tradeoff between happiness and meaning. Global surveys show that when GDP *per capita* goes up, on average, happiness increases at almost the exact same rate as meaning diminishes (Oishi and Diener, 2014). One mechanism that may inform this tradeoff is that when nations do better economically, communal need diminishes, reducing our access to meaning-providing activities. This remarkable stability speaks to the relative nature of our well-being. Still, variance between national communities attests to how some environments, like the Nordic ones, are more conducive to human flourishing. The central role that refugee help has acquired among Norwegian voluntary groups lends support to the claim that Norwegians draw some of their well-being from helping the exceptionally disadvantaged. Research substantiates that exposure to those who are worse off makes people appreciate their own life more (Strack et al., 1990). Our MLS perspective illuminates why such dynamics make meaning-seeking through voluntarism a win-win-win activity, for the beneficiary, altruist, and community as a whole. These virtuous circles of prosociality are among the factors that contribute to why the *World Happiness Report* ranks the Nordic nations 1, 2, 3, 7, and 8—while Poland is 48, Belarus 65, Russia 80, and Ukraine 98 (Helliwell et al., 2022).

### 5.1. Future studies

An MLS perspective offers no magic bullet for policymakers who seek to emulate—or exceed—Nordic success. But our model’s emphasis on prosociality offers some guidance. Good policies are those that pull in the right combination of strings, so that modern prosperity—instead of contributing to misery—can underpin social orders that play along with the idiosyncrasies that our evolutionary past coded into us. To substantiate the utility of this model, we have explored the sources of meaning and their connection to well-being among dedicated altruists. The strength of our study lies in synthesizing the existing evolutionary work on well-being under an MLS umbrella. Our qualitative interviews confirm that people draw well-being from happiness and meaning in the manner that our model predicts. To further support our hypotheses and illuminate underlying mechanisms, more work must be done. The limitations to a qualitative study within a European setting are significant. Large-scale cross-cultural studies would be invaluable for further substantiating what we hypothesize to be human universals with regard to well-being. Quantitative studies could shed light on the importance of temperament and life factors in regard to altruistic motivation and benefit. Qualitative studies could illuminate other forms of prosociality than refugee help. Studies of non-Western cultures should offer fertile contributions to this research. In terms of expanding one’s circles of empathy, we expect there to be significant variance in scale of humanitarian efforts and their narrative justification. Studying non-WEIRD nations and kinship societies could help us better understand the biocultural influences that inform our boundaries for whom to help.

In particular, the happiness part of our equation needs further studies. How its cooperative sources interact and conflict with the meaning side of the equation is challenging to predict. The adaptive benefits of sociality and indirect fitness incentivize exclusion in order to prioritize our inner circles. During crisis, the extent to which one’s resources should be allocated to family and friends versus the larger community makes for demanding calculation. How such concerns influence individual and group well-being might be suitably explored from an MLS perspective. During good times, too, how individuals decide to allocate resources to gain happiness as juxtaposed with meaning is a fruitful research question. Nesse’s claim, that high well-being is a result of adaptive signal interpretation and goal adjustment, needs further substantiation. Qualitative studies of people who have gone through crisis and goal reevaluation could help illuminate the mechanisms of such Nessean flourishing. The stakes are significant. Given how imperative global cooperation will be in the decades ahead and well-being’s importance for motivating prosociality, an evolutionary model with cross-cultural predictive power could be a valuable tool for policymakers who seek new ways to sustain our fraying communities and the planet.

## Data availability statement

The original contributions presented in the study are included in the article/supplementary material, further inquiries can be directed to the corresponding author.

## Ethics statement

Ethical review and approval was not required for the study on human participants in accordance with the local legislation and institutional requirements. The patients/participants provided their written informed consent to participate in this study.

## Author contributions

ML and NW conceptualized, conducted interviews, and edited the manuscript. ML drafted the manuscript. JY conceptualized, analyzed data, and drafted the quantitative study. All authors contributed to the article and approved the submitted version.

## Funding

 This work was conducted as part of the multinational, mixed-method “Grieg” project, which is supported by a European Economic Area grant (project number 2019/34/H/HS6/00597).

## Conflict of interest

The authors declare that the research was conducted in the absence of any commercial or financial relationships that could be construed as a potential conflict of interest.

## Publisher’s note

All claims expressed in this article are solely those of the authors and do not necessarily represent those of their affiliated organizations, or those of the publisher, the editors and the reviewers. Any product that may be evaluated in this article, or claim that may be made by its manufacturer, is not guaranteed or endorsed by the publisher.

## Footnotes

^1^Kahneman et al. (1999) estimated this income threshold to be around $10,000.

^2^Hitokoto and Uchida (2015) define interdependent happiness to encompass "the state of being relationally orientated, preserving quiescence, and embedding oneself in the nexus of ordinariness among others."

^3^Habermas (1997) attempted a similar intervention, to craft a culturally objective cosmopolitanism—termed "legal order"—by grounding it in human rights. This proved misguided; Habermas (2020) offers "postmetaphysical reason" as his culturally sensitive cosmopolitanism.

^4^Hill and Buss (2008) note how one finding seems to contradict that happiness derives from solving adaptively relevant challenges: how having children reduces marital happiness. They suggest several possible proximate causes to this conundrum, for instance, that modern couples lack a large network of extended kin.

^5^Evolutionary scholars have researched what makes humans flourish or not, for instance Gluckman and Hanson (2006), but rarely within the field of positive psychology.

^6^That those affects we term "meaning" evolved to strengthen communities does not entail that all meaning-generating activities fulfill this function. Our well-being system cannot be relied on for consistency and precision. People can be culturally misaligned and ideals can be corrupted.

^7^Norway placed first on the UNDP’s Human Development Index every year from 2001 to 2019, except in 2007 and 2008 when Iceland placed first. Norway placed second in 2020, behind Switzerland (UNDP, 2022).

^8^Not all cooperation is prosocial. Oxford Reference defines "prosocial behavior" to be helping, altruistic, or meant to promote the interests of society, https://www.oxfordreference.com/view/10.1093/oi/authority.20110803100350224. Some evolutionary scholars use the term to denote "the social acceptance of all people. Ultimately, Prosocial is an entire worldview," https://www.prosocial.world.

^9^The power of meaning is attested to by *devoted actor theory*, which accounts for how powerful of a hold meaning quests can have on, for instance, terrorists, and revolutionaries (Atran et al., 2014).

^10^An interesting contradiction to this is how the US is the world’s most "individualistic" country (Hofstede Insights, 2019), yet also has among the highest rates of voluntarism (Anheier and Salamon, 1999).

^11^Social dilemma experiments have generated valuable insights. Lara Aknin is among the scholars who pursue this approach to prosociality and well-being.

^12^"Slavonic" denotes Eastern European countries with Slavic languages. This is a diverse group in terms of relations to and affinity with Russia.

^13^Weird, Educated, Industrialized, Rich, Democratic.
