# Corrigendum: A multilevel selection model for prosocial well-being

**DOI:** 10.3389/fpsyg.2023.1264742

**Published:** 2023-08-01

**Authors:** Mads Larsen, Nina Witoszek, June Chun Yeung

**Affiliations:** ^1^Centre for Development and the Environment, University of Oslo, Oslo, Norway; ^2^Institute of Psychology, Polish Academy of Sciences, Warsaw, Poland

**Keywords:** happiness, meaning, multilevel selection, Nordic model, prosociality, qualitative interviews, volunteerism, well-being societies

In the published article, there was an error in the Funding statement. The grant award number was stated as “103194100” but should be “2019/34/H/HS6/00597.” The correct Funding statement appears below.

